# Gut microbiome of native Arab Kuwaitis

**DOI:** 10.1186/s13099-020-00351-y

**Published:** 2020-02-26

**Authors:** Erica Plummer, Dieter Bulach, Glen Carter, M. John Albert

**Affiliations:** 1 Department of Molecular Microbiology, Murdoch Children’s Research Institute, Melbourne, VIC Australia; 2 Microbiological Diagnostic Unit Public Health Laboratory, The Peter Doherty Institute for Infection and Immunity, The University of Melbourne, Melbourne, VIC Australia; 3 Melbourne Bioinformatics, The University of Melbourne, Carlton, VIC Australia; 4 Department of Microbiology, Faculty of Medicine, Kuwait University, Jabriya, Kuwait

**Keywords:** Gut microbiome, Arab Kuwaiti, 16S rRNA gene, *Bacteroides*, Diet

## Abstract

**Background:**

The human gut microbiome has an important role in health and disease. There is extensive geographical variation in the composition of the gut microbiome, however, little is known about the gut microbiome composition of people from the Arabian Peninsula. In this study, we describe the gut microbiome of Arab Kuwaitis. The gut microbiome of 25 native adult Arab Kuwaitis was characterised using 16S rRNA gene sequencing of the V3–V4 regions. Sequencing data were analysed using DADA2. Phylogeny analysis was performed using amplicon sequence variants (ASVs) assigned to the *Bacteroides* genus and 16S rRNA sequences of *Bacteroides* type strains to understand the relationships among *Bacteroides* ASVs.

**Results:**

About 63% of participants were overweight/obese reflecting normal Kuwaiti population. Firmicutes and Bacteroidetes were the dominant phyla detected in the gut microbiome (representing 48% and 46% of total sequencing reads respectively). At the genus level, *Bacteroides* was the most abundant genus in 22 of 25 participants. A total of 223 ASVs were assigned to the *Bacteroides* genus, eleven of which were present in 50% or more of study participants, reflecting a high diversity of this genus. Phylogenetic analysis revealed that the *Bacteroides dorei/vulgatus* group was the most abundant phylogenetic group (representing 11.91% of all sequence reads) and was detected in all 25 individuals.

**Conclusions:**

*Bacteroides* was the most abundant genus in the gut microbiome of native Arab Kuwaiti adults, with *Bacteroides dorei/vulgatus* forming the predominant phylogenetic group. The microbiome composition would also have been influenced by the nutritional status of participants.

## Introduction

The human gastrointestinal tract is home to trillions of bacteria [1]. Gut microbes play an important role in digestion of nutrients [2], metabolism of drugs and other compounds [3, 4], and synthesis of micronutrients [5], neurotransmitters and other metabolites [6, 7], as well as in the development of gut-specific immune system [8]. The gut microbiome also has an important role in maintaining health, and dysbiosis of the gut microbiome has been associated with various health disorders including metabolic syndrome, diabetes mellitus, obesity, inflammatory bowel disease, colorectal cancer, rheumatoid arthritis, atopy, eczema, autoimmune diseases and psychiatric disorders [9, 10].

There is a high degree of variation in the gut microbiome composition between individuals and within individuals over time, and people living in different geographical regions tend to have different gut microbiome [11, 12]. The diversity and composition of the gut microbiome is influenced by a variety of different factors including diet, environment, antibiotic use, proximity of hosts and host genetics [13, 14] and it is thought that globally, the gut microbiome of healthy individuals segregates into two or three distinct enterotypes based on the abundance of key bacteria—*Bacteroides*, *Prevotella* and members of the Clostridiales order [14, 15].

Characterising the ‘normal’ gut microbiome in different geographical regions provides a comprehensive picture of the microbiome from a global perspective. This is an important baseline information for understanding dysbiosis of the gut microbiome and how it relates to different populations. There are numerous studies on the gut microbiome of populations from various parts of the world [12, 16–22], but only two studies from the Arabian Peninsula [23, 24], a major geographical region. These two studies are from Saudi Arabia. There are no studies from Kuwait, another country in the Arabian Peninsula. There is genetic variation [25] and variation in food consumption patterns across the Arabian Peninsula [26], which would influence the gut microbiome. Therefore, we studied the gut microbiome of native adult Arab Kuwaitis.

## Methods

### Participants

Healthy volunteers, 18 years or older, born and brought up in Kuwait, and of native Arab Kuwaiti ethnicity were recruited for the study. People with a history of intake of probiotics or antibiotics for the past six months, symptoms pertaining to gastrointestinal tract, hypertension, atopic disease, mental illness and high-risk sexual behaviour were excluded.

Body mass index (BMI) was calculated for participants, and was categorized according to the World Health Organization standard categories: underweight < 18.5; normal weight 18.5–24.9; overweight 25.0–29.9; obese ≥ 30 [27].

Participants were presented with a description of a typical Kuwait diet and asked whether their dietary habits agreed with the typical diet or not. Briefly, the Kuwaiti diet consists of meats, dairy products, grains, legumes, vegetables (including leafy greens and herbs), fruits and nuts. A food frequency questionnaire estimated the average daily servings for Kuwaiti adults as 1.9 servings of meat, 3.4 servings of dairy products, 5.3 servings of cereals/cereal products, 3.2 servings of vegetables and 2.8 servings of fruits [28].

### Sample collection and laboratory methods

Participants were asked to provide a fresh faecal sample for microbiome analysis. Faecal specimens were collected by participants into sterile stool containers and transported in a cold box with ice-packs to the laboratory where they were frozen at − 80 °C within 2 h of collection and stored until further analysis. DNA was extracted from stored faecal samples using the QIAamp Fast DNA Stool mini kit as previously described [29]. Dried DNA samples were shipped at ambient temperature to Melbourne, Australia for further analysis. PCR amplification of the variable region V3–V4 of the 16S rRNA gene (341F/805R) [30] was performed followed by amplicon sequencing on the Illumina MiSeq platform (Microbiological Diagnostic Unit, The Peter Doherty Institute for Infection and Immunity, The University of Melbourne; and Micromon, Monash University, Victoria, Australia). A blank negative control was processed and sequenced in the same manner described above to allow for identification of reagent contaminants.

### Sequence and data analysis

Primers were trimmed from demultiplexed reads using TagCleaner [31] and the trimmed sequences were processed using the DADA2 pipeline, version 1.6.0 [32]. Quality control, error rate learning and inference of amplicon sequence variants (ASVs) were performed separately for each of the two sequencing runs to account for run-specific error profiles. Following merging of forward and reverse reads, the two runs were merged into a single sequence table. Chimeras were removed from the merged sequence table and taxonomy was assigned using the DADA2 implementation of the RDP Naive Bayesian Classifier and the Silva reference database (v128). Species level assignment was performed using exact matching in the DADA2 pipeline, allowing for multiple matches per ASV. The sequence table was filtered for contaminants identified in the negative control sample and ASVs were removed if they were present at a relative abundance of 0.001% or less (i.e. fewer than 96 reads).

QIIME 1.9.0 was used to generate rarefaction plots and to determine the core microbiome of specimens, which in this study was defined as ASVs present in at least 85% of specimens. A heatmap was generated by hierarchical clustering of Bray–Curtis dissimilarity distances with Ward’s linkage using the vegan [33] and gplots [34] packages and R v3.4.3 using R studio. Alpha and beta diversity metrics were calculated at ASV level using the vegan package [33]; alpha diversity was calculated using the Shannon diversity index and beta diversity was calculated using the Bray–Curtis index.

The Wilcoxon rank-sum test was used to assess differences in Shannon diversity between microbiome groups as well as assess differences in the Firmicutes: Bacteroidetes ratio of the gut microbiome between individuals with normal BMI compared to individuals with overweight or obese BMI. Statistical analyses were performed using Stata/IC (Version 14.2, StataCorp LP, College Station, USA).

Information relating to the genus *Bacteroides* and the type strains for each of the species, including accession numbers for the 16S rRNA gene, was obtained from bacterio.net (List of Prokaryotic names with Standing in Nomenclature [35]). Reference sequences were trimmed to include only the V3–V4 region of the 16S rRNA gene prior to the production of the multiple sequence alignment of all *Bacteroides* ASVs (i.e. including those present prior to filtering of the ASV table) and type strain sequences. Multiple sequence alignment and inference of the distance tree was performed using Clustal Omega [36]. The tree was used to group related ASVs and classify these phylogeny groups with names based on the type strain taxa included in the group.

The sequence data from this study were deposited in the NCBI Sequence Read Archive (SRA) under project accession number, PRJNA554702.

## Results

Twenty-five participants (10 males and 15 females, Table 1), aged 24 to 57 years were recruited from March 2017 to May 2017 (Table 1). The body mass index (BMI) value was not available for one participant. Ten (41.6%) and five (20.8%) of the remaining 24 participants were overweight and obese respectively. One participant was diagnosed with Crohn’s disease post specimen collection and informed the study team of the diagnosis, but did not report gastrointestinal symptoms at the time of specimen collection. All participants reported consuming a typical Kuwaiti diet.

**Table 1:** Participant characteristics

Unique ID	Age	Gender	BMI	BMI category	Shannon diversity index	Ratio of Firmicutes to Bacteroidetes
KW16S_01	24	F	20.6	Normal	3.43	0.74
KW16S_02	25	F	23.2	Normal	3.54	0.83
KW16S_03	28	M	27	Overweight	3.82	0.78
KW16S_04	44	F	28.3	Overweight	3.22	0.51
KW16S_05	45	F	21.8	Normal	3.39	0.66
KW16S_06	41	M	25.7	Overweight	4.75	1.86
KW16S_07	35	M	29.4	Overweight	2.93	0.29
KW16S_08^a^	57	F			3.04	0.72
KW16S_09	37	M	29.1	Overweight	3.93	0.57
KW16S_10	41	F	25.9	Overweight	4.58	1.27
KW16S_11	27	F	18.6	Normal	5.06	2.93
KW16S_12	44	M	22.6	Normal	4.10	0.98
KW16S_13	30	M	30	Obese	4.54	2.11
KW16S_14	31	M	18.7	Normal	3.78	0.61
KW16S_15	26	F	38.9	Obese	3.86	0.81
KW16S_16	39	F	41.1	Obese	3.96	0.55
KW16S_17	31	F	28.1	Overweight	4.03	0.81
KW16S_18	36	F	21.1	Normal	3.36	0.72
KW16S_19	32	M	41	Obese	3.44	1.35
KW16S_20	43	F	25.3	Overweight	4.04	0.83
KW16S_21	40	F	28.5	Overweight	3.93	0.61
KW16S_22	42	M	26.1	Overweight	3.00	0.56
KW16S_23	38	F	23.4	Normal	4.34	1.47
KW16S_24	52	M	23	Normal	4.83	2.75
KW16S_25	24	F	41.6	Obese	3.95	0.54

*BMI* body mass index

^a^BMI value missing for this participant

A total of 9,420,376 2 × 300 base pair reads were generated from sequencing. Following filtering of the ASV table, 9,360,113 sequences (median of 385,526 reads per sample [interquartile range (IQR) = 128,030–537,590]) representing 1621 ASVs were included in the final analysis. Sequencing data were not rarefied as the rarefaction curves (Additional file 1: Figure S1) which confirmed that specimens had been adequately sampled.

**Fig. 1:**
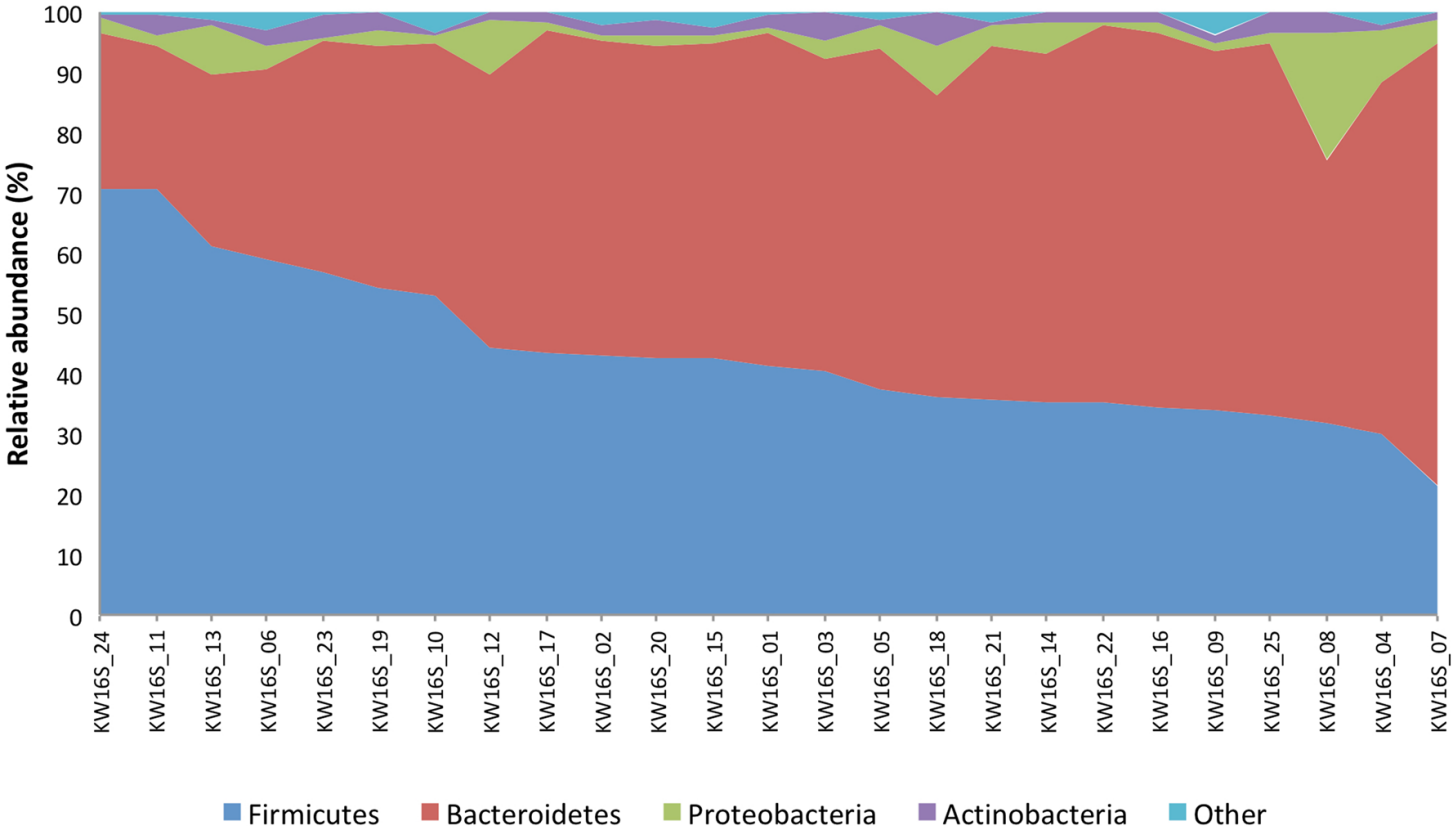
Relative abundance of the gut microbiome at the phylum level. Area chart shows the gut microbiome profiles of study participants

**Fig. 2:**
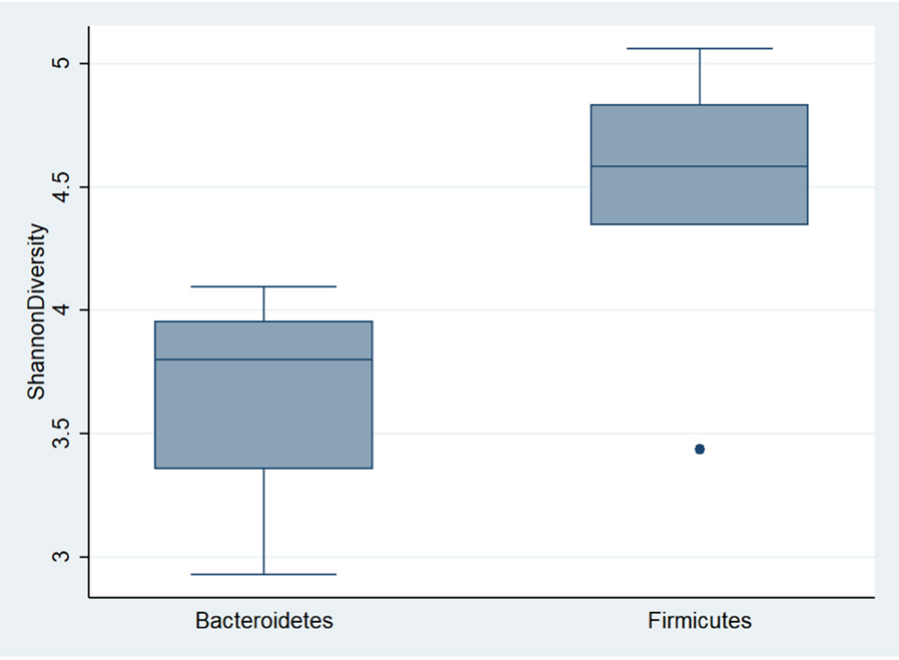
Relationship between dominant phyla and bacterial diversity. Individuals with a Firmicutes dominated gut microbiome had a higher bacterial diversity (as measured by the Shannon diversity index) compared to individuals with a Bacteroidetes dominated microbiome (Wilcoxon rank-sum test statistic = − 3.15, P = 0.002)

Eleven bacterial phyla were detected (Fig. 1). The dominant phyla were Firmicutes and Bacteroidetes (representing 48% and 46% of total sequencing reads respectively), and Bacteroidetes was the most abundant phylum detected in the majority of participants (n = 18/25, 72%). Firmicutes was the most abundant phylum in seven other participants. Interestingly, participants with a gut microbiome abundant in Bacteroidetes had a lower bacterial diversity compared to participants with a microbiome abundant in Firmicutes (median Shannon diversity index in Bacteroidetes abundant gut = 3.80 [IQR = 3.36–3.95] vs. Firmicutes abundant = 4.58 [IQR = 4.34–4.83]; Z = − 3.15, P = 0.002; Fig. 2).

**Fig. 3:**
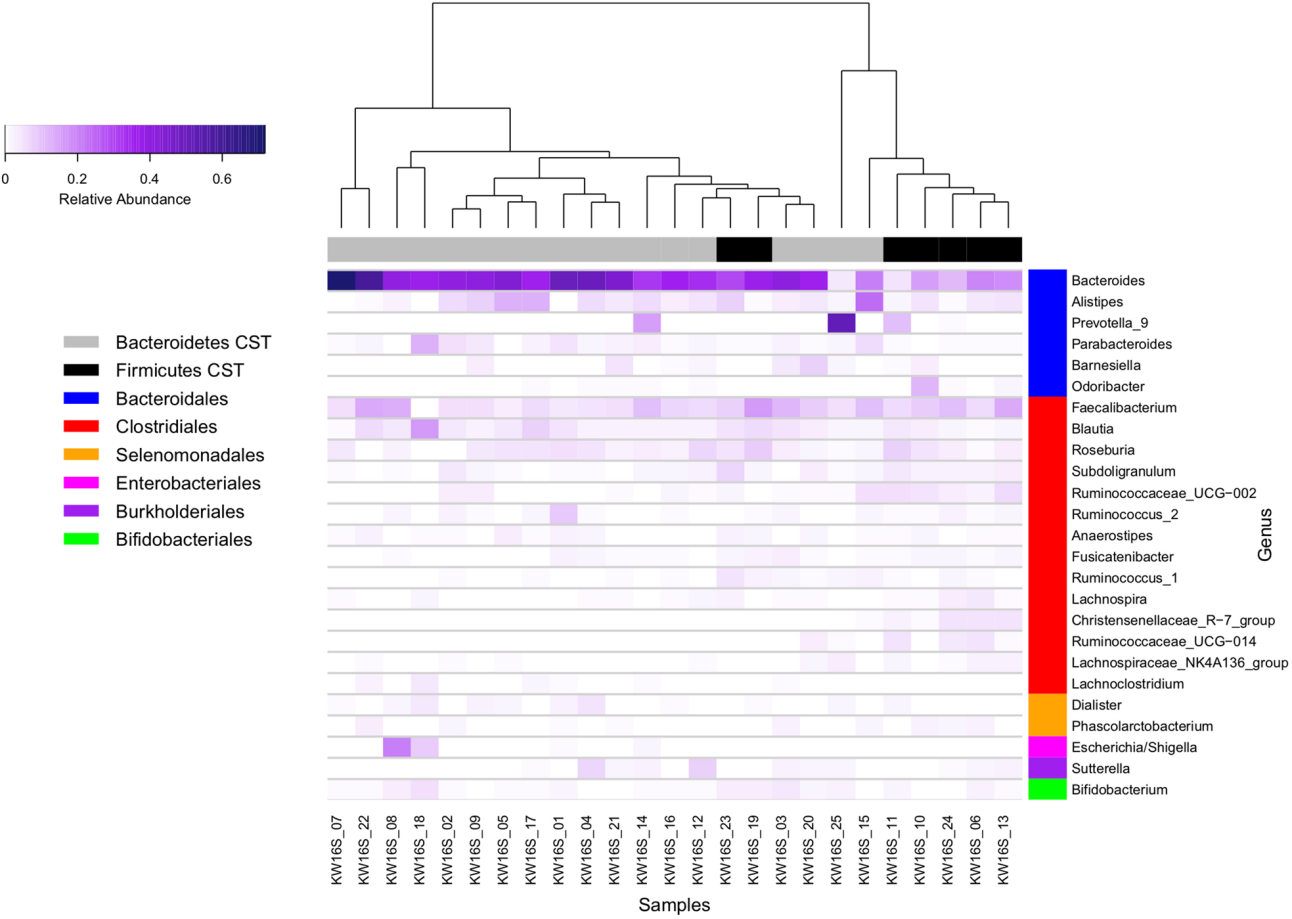
Heatmap of the gut microbiome of native adult Arab Kuwaitis. The heatmap displays the relative abundance of the 25 most abundant bacteria detected in participants in this study. Dominant phylum is displayed above the heatmap in grey (Bacteroidetes dominated microbiome) and black (Firmicutes dominated microbiome). Bacterial order groups are displayed on the right side of the heatmap in blue (Bacteroidales), red (Clostridiales), orange (Selenomonadales), pink (Enterobacteriales), purple (Burkholderiales) and green (Bifidobacteriales). Sample KW16S_15 shown at the bottom of the figure was from the participant who was diagnosed with Crohn’s disease after stool collection. The only discerning feature in this participant was the highest abundance of Alistipes compared to in other participants

At the genus level, specimens clustered into two distinct groups driven by the abundance of *Bacteroides* (Fig. 3), with specimens high in *Bacteroides* clustering together and specimens with lower *Bacteroides* abundance clustering together. *Bacteroides* was detected in all specimens and was the most abundant genus in the majority of participants (n = 22/25, 88%, median relative abundance of 37% [IQR 20–41%]). Of the remaining three participants, one had a gut microbiome dominated by *Prevotella_9* (52% relative abundance), one had a highly diverse microbiome with *Prevotella_9* being the most abundant genus (10% relative abundance) and the third had a high abundance of *Alistipes* relative to other participants (23% vs. median abundance of 4% [range 0–13%]). Interestingly, this participant was the individual diagnosed with Crohn’s disease.

The core gut microbiome of participants was represented by 18 ASVs (Additional file 2: Table S1). Fifteen ASVs representing the core gut microbiome were from the Clostridiales order, primarily the *Lachnospiraceae* and *Ruminococcaceae* families. ASVs representing *Bacteroides fragilis/xylanisolvens* (ASV46), *Bifidobacterium longum* (ASV50) and *Escherichia*/*Shigella* (ASV53) were also identified as members of the core microbiome.

*Bacteroides* ASVs comprised 30% of all sequence reads. Prior to filtering of the ASV table we identified a total of 223 ASVs assigned to the *Bacteroides* genus (113 remained post filtering of the ASV table), eleven of which were present in 50% or more of study participants, indicating a high diversity in *Bacteroides* spp. across individuals. Twenty-three of the 223 identified *Bacteroides* ASVs had an average relative abundance greater than 0.5% and represented more than 80% of the relative abundance of all the *Bacteroides* ASVs (Additional file 3: Table S2). Given the importance of *Bacteroides* spp. in the gut microbiome and the high diversity within this genus between individuals, we investigated this genus further. We produced a phylogeny from a multiple alignment of the 223 *Bacteroides* ASVs and published 16S rRNA gene sequence for each type strain in the *Bacteroides* genus (Additional file 4: Figure S2). The relationships shown in the tree were used to group related ASVs and classify them into phylogeny groups based on the type strain present in the group. Based on the tree, ASV46*-Bacteroides fragilis/xylanisolvens* (which is a part of the core gut microbiome) was more closely related to *B. xylanisolvens/acidofaciens/caecimuris* type strains than *B. fragilis* type strain. The *Bacteroides dorei/vulgatus* group was the most abundant phylogeny group (representing 11.91% of all sequence reads) and was detected in all samples, whereas the *Bacteroides fragilis* group represented 0.73% of total reads and was detected in only 12 of the 25 samples (Additional file 5: Table S3).

Information about BMI was available for 24 participants; nine participants had a BMI of normal, ten had a BMI of overweight and five were considered obese (Table 1). There was no observed difference in the ratio of Firmicutes: Bacteroidetes between individuals with a normal BMI and overweight or obese individuals (median Firmicutes: Bacteroidetes ratio in normal BMI = 0.83 [IQR = 0.72–1.47] vs. overweight/obese = 0.78 [IQR = 0.55–1.27]; P = 0.1440).

## Discussion

It is well established that Firmicutes and Bacteroidetes typically dominate the gut microbiome of healthy adults across the world [37]. This is consistent with our finding that the gut microbiome of adult native Arab Kuwaitis is dominated by either Firmicutes or Bacteroidetes. Individuals with a Firmicutes dominated gut had an increased bacterial diversity compared to those with a Bacteroidetes dominated gut, and *Bacteroides* was the most abundant genus in all but three individuals.

The limited data describing the gut microbiome of people in the Arabian Peninsula has focused on individuals from Saudi Arabia [23, 24], and describes the dominant phyla to be Firmicutes and Actinobacteria. Diet is thought to be a major driver of differences in the gut microbiome composition seen globally [14, 38], and the high abundance of Bacteroidetes in our cohort compared to previous studies of Arabian Peninsula inhabitants could be a result of differences in diet between Kuwaitis and Saudi Arabians. The Kuwaiti diet is generally considered a blend of Arabian, Persian, Indian and Mediterranean cuisines. However, Arabian and Western fast foods rich in meat, sugar and fat are becoming increasingly popular with a consequent rise in the prevalence of obesity, diabetes and hypertension [39]. *Bacteroides* abundance has been associated with Westernized diets high in animal fat and protein [14], and a previous study demonstrated that introduction of a four-day animal based diet resulted in decreased abundance of Firmicutes in the gut microbiome and a subsequent increase in the abundance of spp. from the *Bacteroides*, *Alistipes* and *Bilophilia* genera [40]. It is possible that the high relative abundance of Bacteroidetes observed in our cohort is due to the increasing popularity of Westernized diets among Kuwaitis; however larger studies with more detailed dietary information are needed to investigate this further. It is worth comparing the Kuwaiti flora with the floras in low- and middle-income countries. The remote hunter-gatherer populations such as the Hadza from Tanzania, Pygmies from Central Africa, Mases from Peru and Amerindians from Venezuela have a high microbial diversity and an enriched taxa consisting of *Prevotella*, *Succinovibrio*, *Treponema*, *Cyanobacteria*, Tenericutes, *Clostridium*, *Catenibacterium*, *Eubacterium*, *Lachnospira* and *Salmonella*. *Prevotella* is thought to enhance the ability to digest and extract valuable nutrition from fibrous plant foods. People living in traditional farming or fishing communities like the Bantus of Africa, the Tunapuco population of the Andean highlands or the rural Malawian communities have a high microbial diversity and an enriched taxa consisting of *Prevotella*, *Succinovibrio*, *Treponema*, *Ruminococcus* and *Bacteroides*. This microbiome in traditional communities is thought to exhibit enrichment in carbohydrate- and xenobiotic-processing due to their access to more digestible sugars and therapeutic drugs (summarized in 38). In India, the gut microbiome is dominated by Firmicutes followed by Bacteroidetes, Actinobacteria and Proteobacteria and is enriched with microbial xenobiotic degradation pathways [41].

In China, the abundant taxa are Bacteroidetes, Firmicutes, Proteobacteria and Actinobacteria. However, there was a rural–urban divide with a significantly higher abundance of Proteobacteria in rural population. Urbanization was associated with loss of microbial diversity. Gene diversity increased with urbanization, along with an increase in antibiotic resistance and virulence genes, which were strongly correlated with the presence of *Escherichia* and *Shigella* [42]. In Argentina, *Bacteroides* was the predominant flora [43].

In all these studies, dietary habits were the main driver of microbial composition. Thus, the gut microbiomes of Kuwaitis and Argentines were similar with the predominance of *Bacteroides* in both populations.

The gut microbiome is thought to differ between lean and obese individuals, particularly with respect to the relative abundance of Firmicutes and Bacteroidetes [44]. In a recent cross-sectional study of nearly 4000 adult Kuwaitis, 37% were overweight and 40.3% were obese [45]. Thus, our study participants reflected this general population, as a majority of the participants were overweight/obese. A recent systematic review noted that while several studies have identified obese individuals have a high Firmicutes: Bacteroidetes ratio compared to non-obese individuals, other studies have found contradictory results or no difference in the abundance of these phyla between obese and non-obese individuals [46]. Even though, a majority of our participants were overweight/obese, we did not observe any relationship between BMI and Firmicutes: Bacteroidetes ratio, which may be due to the small sample size. Clavel and Ecker [47] noted that a sample size of at least 500 specimens may be needed to examine associations between BMI and gut microbiome composition. Additionally, comparisons of the Firmicutes: Bacteroidetes ratio offers limited resolution given the numerous species present in these phyla.

Bacteroidetes is a complex phylum, and the class Bacteroidia is made up of five bacterial families (*Bacteroidaceae*, *Marinilabiliaceae*, *Porphyromonadaceae*, *Prevotellaceae* and *Rickenellaceae*), each of which is commonly found in the human gut. *Bacteroides* is one of the six genera under *Bacteroidaceae* and is a highly diverse genus, made up of numerous species (bacterio.net, List of Prokaryotic names with Standing in Nomenclature [35]). *Bacteroides* was the most prevalent and abundant genus identified in our cohort. *Bacteroides* spp. exist in a mutualistic relationship with the human host [48] and have an important role in fermentation of complex sugars [49], metabolism of proteins [50] and deconjugation of bile salts [51]. Bacteroidetes are enriched in patients suffering from type 1 and type 2 diabetes mellitus [52]. A recent study examining the bacterial richness of the gut microbiome of obese and lean individuals found reduced richness to be associated with adiposity, insulin resistance, dyslipidemia and an inflammatory phenotype [53]. Furthermore, participants with reduced bacterial richness had enriched *Bacteroides* spp. [52]. An enriched *Bacteroides* microbiome may be a marker of these characteristics in the Kuwaiti population.

Our phylogenetic analysis of *Bacteroides* revealed a high genetic diversity within individual species and suggests that species from the *Bacteroides dorei/vulgatus* phylogeny group may represent core gut microbiome in this Kuwaiti population. The high diversity of *Bacteroides* spp. across individuals has been observed in other populations [54] and may reflect differences in diets between participants; however, our small sample size prevented us from investigating this further. It is also possible that different *Bacteroides* ASVs present in a phylogeny group may have differing metabolic capabilities.

This study has limitations. It was difficult to recruit participants as obtaining a stool sample from a healthy person is a cultural taboo, thus the sample size was small. However, despite the small sample size, this study presents the first description of the gut microbiome composition of native Arab Kuwaiti adults. While all participants reported consuming a typical Kuwaiti diet, we did not collect detailed dietary or behavioural information leading up to specimen collection, which limits our ability to associate unique gut microbiome profiles with specific diets or behaviours. Finally, 16S rRNA gene studies are subject to primer bias, which may limit detection of relevant taxa, and provides low resolution at the species level. However, using an ASV approach provides consistent labels and allows comparison of ASVs across studies.

## Conclusions

Our results showed that Firmicutes and Bacteroidetes were the dominant phyla in the gut microbiome of Arab Kuwaitis. *Bacteroides* was the most abundant genus with *Bacteroides dorei/vulgatus* forming the predominant phylogenetic group. While the predominance of Bacteroidetes may reflect the increasing Westernization of the Kuwaiti diet, larger studies examining the impact of diet and geography on the gut microbiome in the Arabian Peninsula are needed. As a majority of the study participants was overweight/obese, this also, would have contributed to the microbiome composition.

## Supplementary information

**Additional file 1: Figure S1.** Alpha refraction curve of observed species.

**Additional file 2: Table S1.** The core gut microbiome is represented by 18 ASVs.

**Additional file 3: Table S2.*** Bacteroides* ASVs with average relative abundance of more than 0.50%.

**Additional file 4: Figure S2.** Phylogeny tree showing the inferred relationship between the 225 ASVs classified in the genus *Bacteroides* and the 16S rRNA gene sequences from the type strain for each of the recognized species in the genus *Bacteroides*.

**Additional file 5: Table S3.** Abundance and prevalence of *Bacteroides* phylogeny groups.

## Data Availability

Sequence data are available in the NCBI Sequence Read Archive under the project access number, PRJNA554702. All other data are available in the manuscript.
