# Supplementary material for: Gut microbiome of native Arab Kuwaitis
Source: Gut Pathog. 2020 Feb 26;12:10. doi: 10.1186/s13099-020-00351-y (PMC7043038; doi:10.1186/s13099-020-00351-y)
Supplement: Supplementary file 1 — Additional file 1: Figure S1. Alpha refraction curve of observed species. [file 13099_2020_351_MOESM1_ESM.pdf]

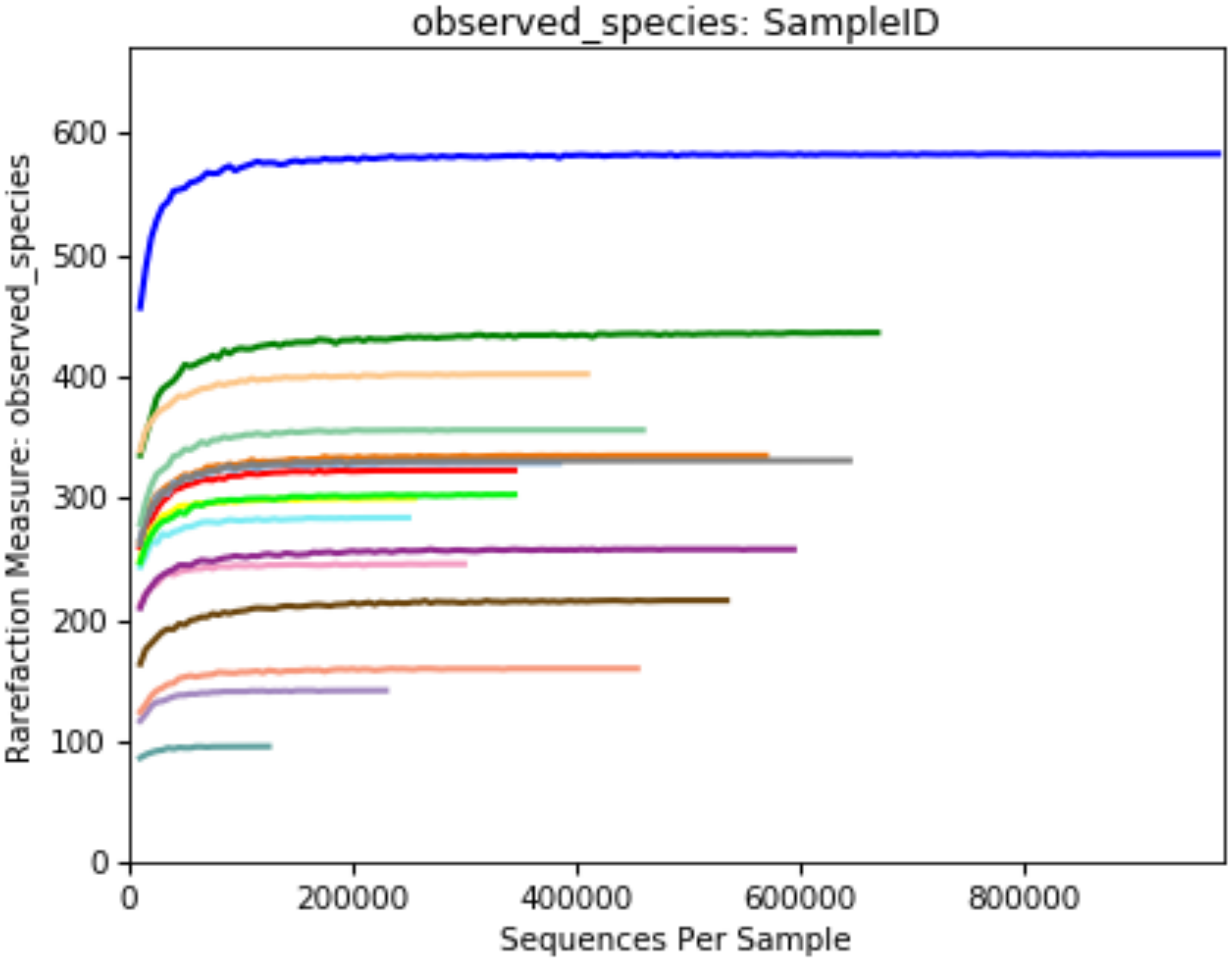

Additional file 1: Figure S1 – Alpha rarefaction curve shows a plateau in the number of observed species (i.e. ASVs) indicating that the samples included in this study were sequenced to an adequate depth.
