# Supplementary material for: Gut microbiome of native Arab Kuwaitis
Source: Gut Pathog. 2020 Feb 26;12:10. doi: 10.1186/s13099-020-00351-y (PMC7043038; doi:10.1186/s13099-020-00351-y)
Supplement: Supplementary file 3 — Additional file 3: Table S2. Bacteroides ASVs with average relative abundance of more than 0.50%. [file 13099_2020_351_MOESM3_ESM.docx]

| **Additional file 3: Table S2 - *Bacteroides* ASVs with average relative abundance >0.50%** | | | | | |
| --- | --- | --- | --- | --- | --- |
| **ASV** | **Genus** | **Species** | **Phylogeny Group^a^** | **Average relative abundance^b^** | **Prevalence^c^** |
| ASV15 | Bacteroides | cellulosilyticus/intestinalis | cellulosilyticus | 0.86 | 14 |
| ASV25 | Bacteroides | NA | clarus | 0.90 | 3 |
| ASV1 | Bacteroides | dorei/vulgatus | dorei/vulgatus | 5.34 | 19 |
| ASV14 | Bacteroides | vulgatus | dorei/vulgatus | 1.97 | 14 |
| ASV9 | Bacteroides | NA | dorei/vulgatus | 1.33 | 1 |
| ASV12 | Bacteroides | vulgatus | dorei/vulgatus | 1.32 | 12 |
| ASV137 | Bacteroides | vulgatus | dorei/vulgatus | 0.76 | 2 |
| ASV45 | Bacteroides | NA | dorei/vulgatus | 0.62 | 7 |
| ASV157 | Bacteroides | vulgatus | dorei/vulgatus | 0.56 | 2 |
| ASV7 | Bacteroides | eggerthii | eggerthii | 1.05 | 7 |
| ASV18 | Bacteroides | NA | eggerthii | 0.60 | 3 |
| ASV23 | Bacteroides | thetaiotaomicron | faecichinchillae | 0.73 | 20 |
| ASV44 | Bacteroides | fragilis | fragilis | 0.73 | 12 |
| ASV99 | Bacteroides | NA | koreensis/kribbi/ovatus | 0.82 | 11 |
| ASV20 | Bacteroides | fragilis/ovatus | koreensis/kribbi/ovatus | 0.80 | 18 |
| ASV22 | Bacteroides | fragilis/ovatus | koreensis/kribbi/ovatus | 0.65 | 16 |
| ASV108 | Bacteroides | fragilis/ovatus | koreensis/kribbi/ovatus | 0.52 | 8 |
| ASV19 | Bacteroides | massiliensis | massiliensis | 0.66 | 10 |
| ASV37 | Bacteroides | plebeius | plebeius | 0.83 | 7 |
| ASV8 | Bacteroides | uniformis | uniformis/rodentium | 2.69 | 16 |
| ASV3 | Bacteroides | uniformis | uniformis/rodentium | 2.60 | 19 |
| ASV46 | Bacteroides | fragilis/xylanisolvens | xylanisolvens/acidofaciens/caecimuris | 0.83 | 22 |
| ASV83 | Bacteroides | ovatus/xylanisolvens | xylanisolvens/acidofaciens/caecimuris | 0.69 | 16 |

^a^ Phylogeny Groups are named based on Supplementary Figure 2 according to the species of the type strain sequence included in the group

^b^ Average relative abundance of each ASV across all 25 specimens

^c^ Prevalence = number of specimens out of 25 in which that ASV was present
