# Supplementary material for: Gut microbiome of native Arab Kuwaitis
Source: Gut Pathog. 2020 Feb 26;12:10. doi: 10.1186/s13099-020-00351-y (PMC7043038; doi:10.1186/s13099-020-00351-y)
Supplement: Supplementary file 4 — Additional file 4: Figure S2. Phylogeny tree showing the inferred relationship between the 225 ASVs classified in the genus Bacteroides and the 16S rRNA gene sequences from the type strain for each of the recognized species in the genus Bacteroides. [file 13099_2020_351_MOESM4_ESM.pdf]

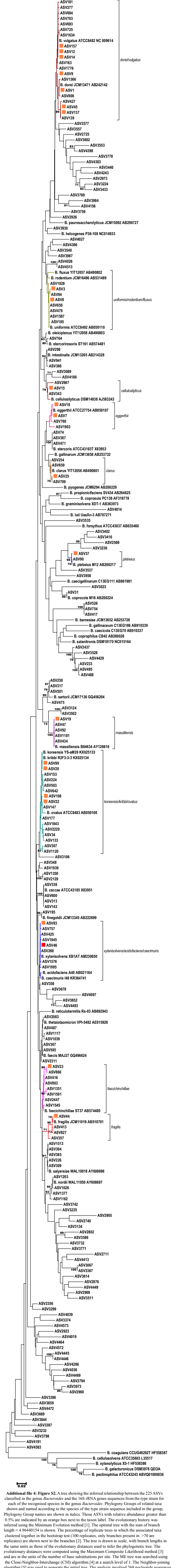

Additional file 4: Figure S2. A tree showing the inferred relationship between the 225 ASVs classified in the genus *Bacteroides* and the 16S rRNA genes sequences from the type strain for each of the recognised species in the genus *Bacteroides*. Phylogeny Groups of related taxa shown and named according to the species of the type strain sequence included in the group, Phylogeny Group names are shown in italics. Those ASVs with relative abundance greater than 0.5% are indicated by an orange box next to the taxon label. The evolutionary history was inferred using the Minimum Evolution method [1]. The optimal tree with the sum of branch length = 4.96440154 is shown. The percentage of replicate trees in which the associated taxa clustered together in the bootstrap test (100 replicates, only branches present in >70 are replicates) are shown next to the branches [2]. The tree is drawn to scale, with branch lengths in the same units as those of the evolutionary distances used to infer the phylogenetic tree. The evolutionary distances were computed using the Maximum Composite Likelihood method [3] and are in the units of the number of base substitutions per site. The ME tree was searched using the Close-Neighbor-Interchange (CNI) algorithm [4] at a search level of 1. The Neighbor-joining algorithm [5] was used to generate the initial tree. The analysis involved 268 nucleotide sequences. All positions containing gaps and missing data were eliminated. There were a total of 388 positions in the final dataset. Evolutionary analyses were conducted in MEGA7 [6].

1. Rzhetsky A. and Nei M. (1992). A simple method for estimating and testing minimum evolution trees. Molecular Biology and Evolution 9:945-967.
2. Felsenstein J. (1985). Confidence limits on phylogenies: An approach using the bootstrap. Evolution 39:783-791.
3. Tamura K., Nei M., and Kumar S. (2004). Prospects for inferring very large phylogenies by using the neighbor-joining method. Proceedings of the National Academy of Sciences (USA) 101:11030-11035.
4. Nei M. and Kumar S. (2000). Molecular Evolution and Phylogenetics. Oxford University Press, New York.
5. Saitou N. and Nei M. (1987). The neighbor-joining method: A new method for reconstructing phylogenetic trees. Molecular Biology and Evolution 4:406-425.
6. Kumar S., Stecher G., and Tamura K. (2016). MEGA7: Molecular Evolutionary Genetics Analysis version 7.0 for bigger datasets. Molecular Biology and Evolution 33:1870-1874.
