# Supplementary material for: Gut microbiome of native Arab Kuwaitis
Source: Gut Pathog. 2020 Feb 26;12:10. doi: 10.1186/s13099-020-00351-y (PMC7043038; doi:10.1186/s13099-020-00351-y)
Supplement: Supplementary file 5 — Additional file 5: Table S3. Abundance and prevalence of Bacteroides phylogeny groups. [file 13099_2020_351_MOESM5_ESM.docx]

| **Additional file 5: Table S3 - Abundance and prevalence of *Bacteroides* phylogeny groups** | | |
| --- | --- | --- |
| **Phylogeny group** | **Average relative abundance^a^** | **Prevalence^b^** |
| *B. cellulosilyticus* | 0.86 | 14 |
| *B. clarus* | 0.90 | 3 |
| *B. dorei/vulgatus* | 11.91 | 25 |
| *B. eggerthii* | 1.65 | 10 |
| *B. faecichinchillae* | 0.73 | 20 |
| *B. fragilis* | 0.73 | 12 |
| *B. koreensis/kribbi/ovatus* | 2.79 | 24 |
| *B. massiliensis* | 0.66 | 10 |
| *B. plebeius* | 0.83 | 7 |
| *B. uniformis/rodentium* | 5.30 | 24 |
| *B. xylanisolvens/acidofaciens/caecimuris* | 1.52 | 22 |

^a^ Average relative abundance of each phylogeny group across all 25 specimens

^b^ Prevalence = number of specimens out of 25 in which that phylogeny group was present
